# Enriched environment ameliorates adult hippocampal neurogenesis deficits in *Tcf4* haploinsufficient mice

**DOI:** 10.1186/s12868-020-00602-3

**Published:** 2020-11-23

**Authors:** Katharina Braun, Benjamin M. Häberle, Marie-Theres Wittmann, D. Chichung Lie

**Affiliations:** 1 Institute of Biochemistry, Emil Fischer Center, Friedrich-Alexander-Universität Erlangen-Nürnberg, 91054 Erlangen, Germany; 2 Institute of Human Genetics, Universitätsklinikum Erlangen, Friedrich-Alexander-Universität Erlangen-Nürnberg, 91054 Erlangen, Germany

**Keywords:** *Tcf4*, PTHS, Adult neurogenesis, Hippocampus, Intellectual disability, Enriched environment

## Abstract

**Background:**

Transcription factor 4 (*TCF4*) has been linked to human neurodevelopmental disorders such as intellectual disability, Pitt-Hopkins Syndrome (PTHS), autism, and schizophrenia. Recent work demonstrated that TCF4 participates in the control of a wide range of neurodevelopmental processes in mammalian nervous system development including neural precursor proliferation, timing of differentiation, migration, dendritogenesis and synapse formation. TCF4 is highly expressed in the adult hippocampal dentate gyrus – one of the few brain regions where neural stem / progenitor cells generate new functional neurons throughout life.

**Results:**

We here investigated whether *TCF4* haploinsufficiency, which in humans causes non-syndromic forms of intellectual disability and PTHS, affects adult hippocampal neurogenesis, a process that is essential for hippocampal plasticity in rodents and potentially in humans. Young adult *Tcf4* heterozygote knockout mice showed a major reduction in the level of adult hippocampal neurogenesis, which was at least in part caused by lower stem/progenitor cell numbers and impaired maturation and survival of adult-generated neurons. Interestingly, housing in an enriched environment was sufficient to enhance maturation and survival of new neurons and to substantially augment neurogenesis levels in *Tcf4* heterozygote knockout mice.

**Conclusion:**

The present findings indicate that haploinsufficiency for the intellectual disability- and PTHS-linked transcription factor TCF4 not only affects embryonic neurodevelopment but impedes neurogenesis in the hippocampus of adult mice. These findings suggest that TCF4 haploinsufficiency may have a negative impact on hippocampal function throughout adulthood by impeding hippocampal neurogenesis.

## Introduction

The transcription factor 4 (TCF4, Gene ID: 6925) forms together with its paralogues TCF3 and TCF12 the class I basic Helix-Loop-Helix (bHLH) subgroup of transcription factors [1]. TCF4 has received growing attention following the discovery that loss-of-function mutations and single nucleotide polymorphisms are linked to neuropsychiatric disease. In humans, the TCF4 gene consists of 20 exons, 18 of which are protein-coding. Heterozygote loss-of-function mutations of *TCF4* before exon 7 have been linked to non-specific intellectual disability [2–4], while heterozygote disrupting mutations after exon 9 have been causally linked to Pitt-Hopkins Syndrome (PTHS, MIM #610954), a neurodevelopmental disorder characterized by distinctive facial features, moderate to severe intellectual disability, autistic behavior, intermittent breathing abnormalities, seizures [5–7]. Moreover, SNPs in non-coding regions of the TCF4 gene are associated with an increased risk of schizophrenia and autism [8–11].

TCF4 is broadly expressed throughout the developing human and murine cortex and hippocampus [12–14]. TCF4 has pleiotropic functions in neurodevelopment. In rodents, TCF4 controls proliferation of cortical precursor cells, balances precursor proliferation versus differentiation, regulates fate choices and migration, and modulates dendrite and spine development [14–18]. Loss of *Tcf4* causes imbalanced generation of deep vs. upper layer neurons, delays neuronal differentiation, and impairs dendritogenesis and synapse formation [14, 17, 19]. Development of the hippocampal formation appears to be particularly reliant on precise TCF4 dosage. MR-analyses uncovered small hippocampi in individuals with PTHS [13, 20]. Heterozygote *Tcf4* knockout mice show a significant reduction in hippocampal volume [13], while homozygote TCF4 knockout mice and transgenic mice with a homozygote embryonic neural stem cell specific-deletion of TCF4 show severe hippocampal hypoplasia [12, 17, 19].

TCF4 is highly expressed in the adult brain [13], and is thought to have critical function in the regulation of neural plasticity [21–23]. High levels of TCF4 expression are observed in the dentate gyrus of the hippocampal formation [13]. The dentate gyrus is one of the few regions of the CNS, where neural stem cells give rise to new neurons throughout adulthood. Adult hippocampal neurogenesis has been unequivocally demonstrated in rodents and non-human primates [24]. Studies using different methodologies to detect adult-born neurons provided strong evidence for the existence of adult hippocampal neurogenesis in humans [25–27]. Nevertheless, the notion of adult neurogenesis in humans remains contested as a recent study failed to detect the expression of markers indicative of neurogenesis in the adult human hippocampus [28]. In rodents, adult hippocampal neurogenesis is critical for the regulation of anxiety and depression-like behaviour as well as for hippocampus-dependent learning and memory [29]. Notably, impaired adult hippocampal neurogenesis was found to contribute to cognitive deficits in preclinical models for autism-spectrum disorders and intellectual disability [30–33].

Here, we sought to determine whether *Tcf4* haploinsufficiency, which has been associated with autism and intellectual disability in humans, affects adult hippocampal neurogenesis. Analysis of *Tcf4* heterozygote knockout mice revealed that *Tcf4* haploinsufficiency is associated with a smaller size of the hippocampal neural stem/progenitor cell pool and impaired maturation and survival of adult-born dentate granule neurons. Interestingly, long-term housing in an enriched environment enhanced survival of adult-born dentate granule neurons and substantially increased adult hippocampal neurogenesis levels, raising the interesting possibility that in mice behavioural modifications during adulthood can ameliorate a subset of neural deficits caused by *TCF4* haploinsufficiency.

## Results

### *Tcf4* haploinsufficiency leads to proliferation deficits in adult neurogenesis

**Fig. 1:**
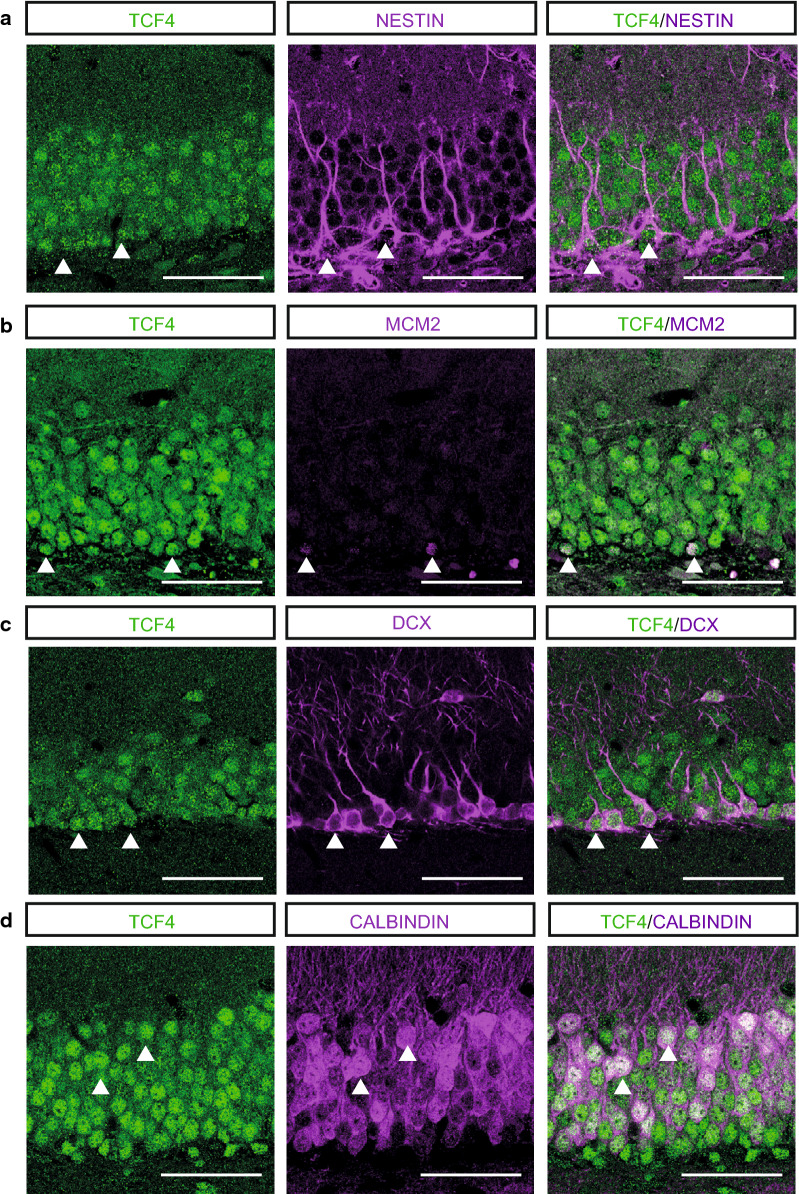
TCF-4 is expressed at all stages of adult neurogenesis. Representative images of TCF-4 and NESTIN **a**, MCM2 **b**, DCX **c** and CALBINDIN **d**. NESTIN is a marker for aNSCs, MCM2 marks proliferative cells. DCX marks immature neurons and CALBINDIN is a marker for mature granule cell neurons. The triangles mark example cells with co-expression of TCF-4 with the respective marker. Scale bar, 50 µm

**Fig. 2:**
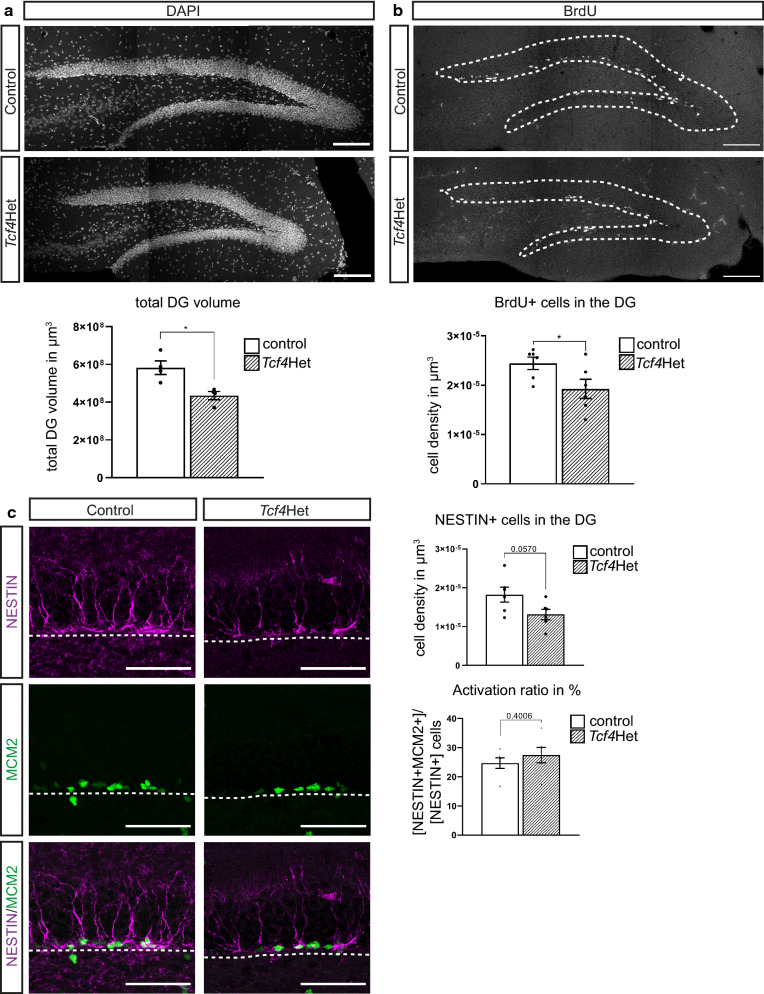
*Tcf4* haploinsufficiency leads to proliferation deficits in adult neurogenesis**. a** Representative images and quantification of the DG volume of control and *Tcf4*Het mice. Haploinsufficient mice show a reduced volume of the DG in comparison to control mice. Scale bar, 100 µm; n = 4. **b** Representative images and quantification of BrdU incorporating cells in the DG 3 h after last BrdU injection. *Tcf4*Het mice have a decreased number of BrdU + cells in DG. Scale bar, 100 µm; n = 6. **c** Representative images and quantification of the number of aNSCs and their activation. *Tcf4*Het animals show a strong trend towards lower number of NESTIN + cells. The activation ratio is unaltered. Scale bar, 50 µm; n = 6. Data are presented as mean ± SEM

We first performed immunohistochemical analysis against TCF4 and select stage-specific markers to confirm the notion that TCF4 is expressed in the adult hippocampal neurogenic lineage. Indeed, TCF4 co-localized with the radial glia like marker NESTIN (Fig. 1a), with MCM2, a marker for proliferating precursor cells (Fig. 1b), with DCX, a marker for immature neurons (Fig. 1c) and with CALBINDIN, a marker for mature granule cells (Fig. 1d), indicating that TCF4 is expressed during all stages of adult hippocampal neurogenesis. In line with the central function of TCF4 in hippocampal development and our previous report, *Tcf4* haploinsufficient mice (*Tcf4Het*) showed a significantly reduced volume of dentate gyrus granule cell layer [Volume in µm^3^: control 5.77 × 10^8^ ± 3.58 × 10^7^; *Tcf4*Het 4.51 × 10^8^ ± 2.41 × 10^7^; p-value = 0.012 (Fig. 2a)] [13].

Next, we investigated whether adult hippocampal neurogenesis is altered by *Tcf4* haploinsufficiency. To assess the impact of *Tcf4* haploinsufficiency on proliferation, young adult *Tcf4Het* and control mice were injected for three consecutive days with the thymidine analogue BrdU and sacrificed three hours after the final injection. *Tcf4*Het mice showed a significant decrease in the number of BrdU positive cells even following correction for the smaller dentate gyrus volume [BrdU + cells/µm^3^: control 2.44 × 10^–5^ ± 1.14 × 10^–6^; *Tcf4*Het 1.93 × 10^–5^ ± 1.79 × 10^–6^; p-value = 0.049 (Fig. 2b)], indicating that *Tcf4* haploinsufficiency reduced proliferation. *Tcf4* haploinsufficient mice showed a strong trend towards lower densities of radial glia-like stem/precursor cells [NESTIN + cells/µm^3^: control 1.82 × 10^–5^ ± 1.94 × 10^–6^; *Tcf4*Het 1.31 × 10^–5^ ± 1.35 × 10^–6^; p-value = 0.0570 (Fig. 2c)]. The fraction of MCM2 + cells amongst the NESTIN + population, however, was comparable [NESTIN + MCM2 + / NTIN + cells in %: control 24.71 ± 1.81; *Tcf4*Het 27.50 ± 2.62; p-value = 0.4006 (Fig. 2c)], suggesting that while *Tcf4* haploinsufficiency resulted in a strong tendency towards lower numbers of radial glia-like stem/precursor cells, it did not affect stem/precursor cell activation. Given that the reduction in cell proliferation was comparable to the reduction in the number of radial glia-like stem/precursor cells, it seems likely that the decreased proliferative activity in the adult neurogenic niche of *Tcf4*Het mice is to a significant extent caused by decreased radial glia-like stem/precursor cell numbers.

### *Tcf4* haploinsufficiency impairs neuronal fate decision, differentiation and maturation in adult neurogenesis

**Fig. 3:**
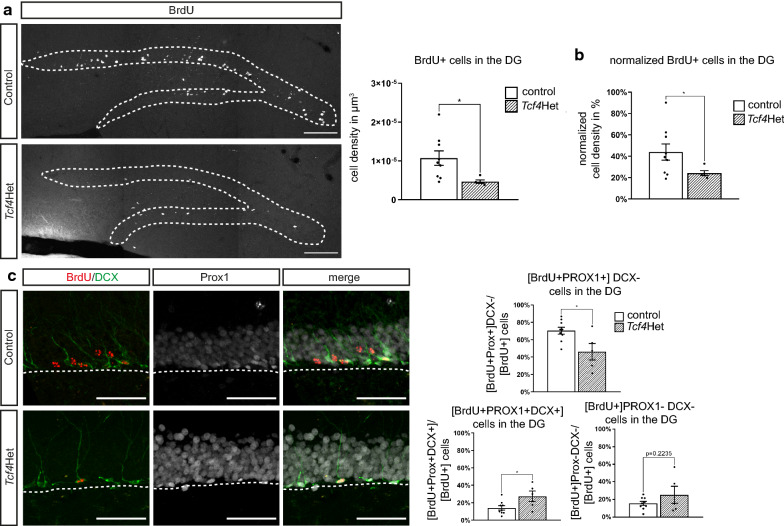
*Tcf4* haploinsufficiency impairs neuronal survival, differentiation and maturation in adult neurogenesis. **a** Representative images and quantification of BrdU incorporating cells in the DG four weeks after last BrdU injection. *Tcf4*Het mice have a decreased number of BrdU + cells in DG. Scale bar, 100 µm; n = 9 and 5. **b** Quantification of the percentage of surviving cells normalized to number of cells that were generated four weeks before. **c** Representative images and quantification of the number of BrdU + single, BrdU + DCX + PROX1 + triple and BrdU + PROX1 + double positive cells four weeks after last BrdU injection. PROX1 labels granule neurons and DCX immature neurons. The number of immature neurons (BrdU + DCX + PROX1 + triple positive) is increased in *Tcf4*Het animals, whereas the number of mature neurons (BrdU + PROX1 + positive) is reduced. Scale bar, 50 µm; n = 9 and 5. Data are presented as mean ± SEM

We next determined the number of BrdU positive cells four weeks after a three-day BrdU pulse. *Tcf4* haploinsufficient mice showed a reduced number of BrdU incorporating cells in the DG of compared to control [BrdU + cells/µm^3^: control 1.07 × 10^–5^ ± 1.75 × 10^–6^; *Tcf4*Het 4.67 × 10^–6^ ± 3.96 × 10^–7^; p-value = 0.011 (Fig. 3a)]. The ratio of the number of BrdU + cells between the four week and three-hour time-point was strongly reduced in *Tcf4*Het mice [BrdU ratio in %: control 43.93 ± 7.63; *Tcf4*Het 24.25 ± 2.3; p-value = 0.034 (Fig. 3b)], indicating that *Tcf4* haploinsufficiency impaired survival of newborn cells.

To evaluate the fate of surviving cells we co-stained BrdU with DCX and PROX1, markers for immature neurons and granule neurons, respectively. The fraction of BrdU + cells expressing a neuronal marker appeared to be slightly reduced in *Tcf4*Het mice. Interestingly, quantification of PROX1 + /DCX + double positive cells (immature neurons) and of PROX1 + /DCX− cells (mature neurons) revealed a significant increase in the fraction of immature neurons [PROX1 + /DCX + amongst BrdU + cells in %: control 13.98 ± 2.40; *Tcf4*Het 27.45 ± 5.55; p-value = 0.034 (Fig. 3c)] and a substantial decrease in the fraction of mature neurons [BrdU + PROX1 + DCX- in %: control 70.37 ± 3.79; *Tcf4*Het 46.17 ± 8.54; p-value = 0.017 (Fig. 3c)] amongst BrdU-labeled cells, suggesting that *Tcf4* haploinsufficiency interfered with the generation of mature dentate granule neurons.

### Enriched environment ameliorates defects in adult neurogenesis due to *Tcf4* haploinsufficiency

**Fig. 4:**
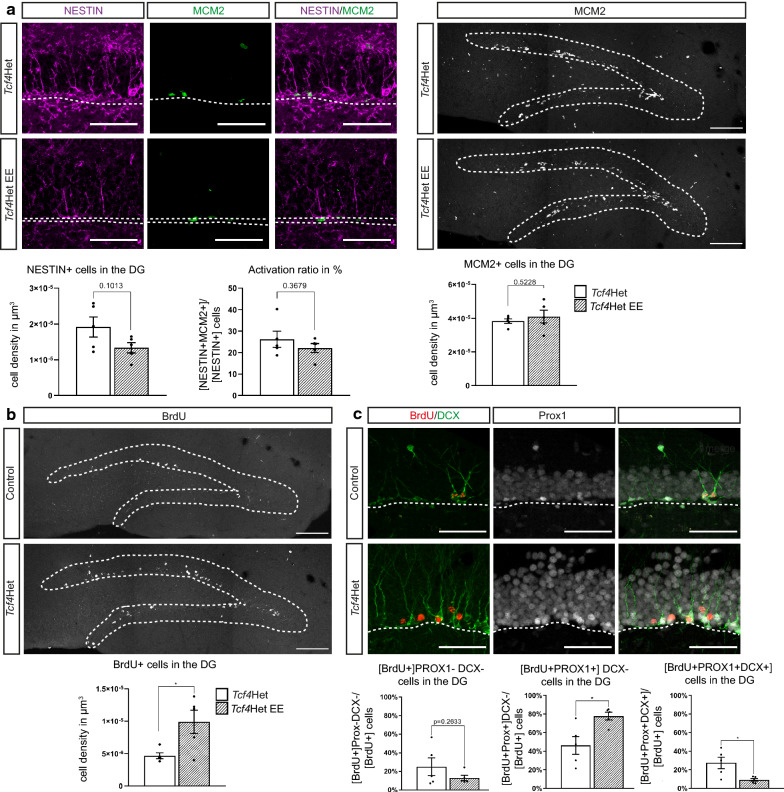
Enriched environment ameliorates defects in adult neurogenesis due to *Tcf4* haploinsufficiency. **a** Representative images and quantification of the number of proliferative cells, NESTIN + (aNSCs) and the activation of aNSCs in the DG after five weeks of EE. The number of proliferative cells, aNSCs and the activation ratio of aNSC is unaltered. Scale bar, 50 µm; n = 5. **b** Representative images and quantification of BrdU incorporating cells in the DG four weeks after last BrdU injection and after five weeks of EE. *Tcf4*Het EE mice have an increased number of BrdU + cells in DG. Scale bar, 100 µm; n = 5. **c** Representative images and quantification of the number of BrdU + single, BrdU + DCX + PROX1 + triple and BrdU + PROX1 + double positive cells four weeks after last BrdU injection and after five weeks of EE. The number of immature neurons (BrdU + DCX + PROX1 + triple positive) is decreased in *Tcf4*Het EE animals, whereas the number of mature neurons (BrdU + PROX1 + positive) is increased. Scale bar, 50 µm; n = 5. Data are presented as mean ± SEM

Enriched environment (EE) is a powerful stimulant of adult hippocampal neurogenesis in adult wildtype mice [34–36]. We next sought to determine, whether EE can be harnessed to ameliorate neurogenesis deficits in *Tcf4* haploinsufficient mice. To this end, we placed a cohort of *Tcf4*Het mice—in parallel to the above described cohorts, which were kept under home cage conditions—in EE conditions (*Tcf4*Het EE). Similar to the other cohorts, animals received a 3-day pulse of BrdU. Analysis of the *Tcf4*Het EE cohort was performed four weeks after the final BrdU injection. Quantification of NESTIN + and MCM2 + cells indicated that EE did not affect radial glia-like cell numbers and their activation [NESTIN + cells/µm^3^: *Tcf4*Het 1.92 × 10^–5^ ± 2.51 × 10^–6^; *Tcf4*Het EE 1.34 × 10^–5^ ± 1.43 × 10^–6^; p-value = 0.1013 (Fig. 4a); NESTIN + MCM2 + / NESTIN + cells in %: *Tcf4*Het 26.23 ± 3.34; *Tcf4*Het EE 22.13 ± 2.11; p-value = 0.1022 (Fig. 4a)], and overall proliferation activity [MCM2 + cells/µm^3^: *Tcf4*Het 3.83 × 10^–5^ ± 1.21 × 10^–6^; *Tcf4*Het EE 4.10 × 10^–5^ ± 3.81 × 10^–6^; p-value = 0.523 (Fig. 4a)].

*Tcf4*Het mice housed in an enriched environment, however, had a twofold increase in BrdU + cells compared to *Tcf4* haploinsufficient mice housed in home cages (*Tcf4*Het) [BrdU + cells/µm^3^: *Tcf4*Het 4.67 × 10^–6^ ± 4.43 × 10^–7^; *Tcf4*Het EE 9.88 × 10^–6^ ± 1.81 × 10^–6^; p-value = 0.0232 (Fig. 4d)]. Notably, EE decreased the fraction of PROX1 + /DCX + immature neurons [PROX1 + DCX + /BrdU + in %: *Tcf4*Het 27.45 ± 5.55; *Tcf4*Het EE 9.13 ± 1.37; p-value = 0.020 (Fig. 4e)] and increased the fraction of PROX1 + /DCX- mature granule neurons amongst BrdU labelled cells [BrdU + PROX1 + DCX- in %: *Tcf4*Het 46.17 ± 8.54; *Tcf4*Het EE 77.74 ± 3.68; p-value = 0.0162 (Fig. 4e)]. Collectively, these data indicate that long-term exposure to EE ameliorates survival and maturation deficits in adult hippocampal neurogenesis of *Tcf4* haploinsufficient mice.

## Discussion

Loss-of-function mutations in the bHLH transcription factor TCF4 are linked to neurodevelopmental disorders such as intellectual disability and Pitt-Hopkins Syndrome. Here, we analysed a *Tcf4* heterozygote knockout mouse model to begin to shed light on the dependency of adult neurogenesis in the hippocampal dentate gyrus on *Tcf4* gene dosage. Our analyses confirm that constitutive *Tcf4* haploinsufficiency is associated with a reduced dentate gyrus size and reveal a profound reduction in the production of new dentate granule neurons during adulthood. We found that *Tcf4* haploinsufficiency reduced the proliferative activity in the adult dentate gyrus. Given that *Tcf4*Het mice showed a 30% reduction in radial-glia like stem / progenitor cells and that the reduction in proliferation (about 20% less BrdU incorporating cells) was in a comparable range, it seems most likely that the proliferation deficit was to a large part the consequence of a smaller radial-glia like stem / progenitor cell pool. It will be interesting to determine, why the *Tcf4* haploinsufficient dentate gyrus harbours a smaller stem cell population. Radial-glia like stem / progenitor cells in the murine dentate gyrus are derived from neural precursor cells in the mouse dentate neuroepithelium, which migrate into the primitive dentate region and enter quiescence around postnatal day 7. TCF4 is highly expressed in the dentate neuroepithelium and the developing hippocampus [12, 13]. Considering recent findings that TCF4 dosage affects proliferation of embryonic neural precursor cells [14–16] and cell migration in the developing CNS [14, 17] including the migration of hippocampal neural progenitors [12], it is tempting to speculate that a combination of proliferation and migration defects of neural precursors contribute to the decreased size of the neural stem/progenitor cell pool in the adult dentate gyrus. TCF4 and the related E-protein TCF3 inhibit neural stem cell differentiation and cell cycle exit in the adult subventricular zone [37] raising the alternative possibility that *Tcf4* haploinsufficiency results in accelerated stem cell depletion due to premature neuronal fate commitment.

*Tcf4* haploinsufficiency had a profound impact on survival of adult generated cells. It is possible that TCF4 directly regulates the expression of, e.g., anti-apoptotic pathways in the adult neurogenic lineage. Adult-born neuron survival is highly dependent on maturation and synaptic integration [38–40]. Recent studies reported that loss-of-*Tcf4* causes delayed maturation of embryonically and early postnatally born neurons, impairs dendrite and synapse formation in the developing cortex [17, 41], and decreases spine density of mature cortical and hippocampal neurons [42]. When analysing the marker profile of 4-week old adult-born neurons, we found that neurons in *Tcf4*Het mice less frequently displayed a mature marker profile, which suggests the possibility that *Tcf4* haploinsufficiency impaired maturation and thereby decreased survival of adult-born neurons.

How TCF4 regulates maturation of neurons on the molecular level remains to be determined. E-box proteins such as TCF4, TCF3 and TCF12 function as transcription factors as homodimers or through formation of heterodimers with bHLH class II transcription factors such as the proneural transcription factors Neurog1 and 2 and the transcription factors of the NeuroD family [1]. NeuroD1 and NeuroD2 have been implied in the maturation of adult-born hippocampal neurons [43, 44] and it will be interesting to test whether their interaction with TCF4 is required for dentate granule neuron maturation.

Given our primary goal to gain first insight how *Tcf4* haploinsufficiency, which has been causally linked to neurodevelopmental disorders such as Pitt-Hopkins Syndrome and intellectual disability, affects adult neurogenesis, we analysed constitutive heterozygote *Tcf4* knockout mice. TCF4 is broadly expressed and is critical for the development of a number of neural and non-neural cell types [18, 45–48]. It therefore remains to be determined how decreased TCF4 dosage in different cell populations contributes to the impairment in adult hippocampal neurogenesis.

We made the interesting observation that long-term exposure to an enriched environment substantially increased the generation of new neurons with a mature marker profile, indicating that behavioural modifications and environmental stimulation may ameliorate TCF4 dosage-dependent defects. Exposure to an enriched environment promotes hippocampal network activity and stimulates adult-born neuron survival and maturation [35, 49]. Interestingly, recent work demonstrated that the function of TCF4 is neuronal activity dependent [18, 50, 51] raising the intriguing possibility that enriched environment ameliorated hippocampal neurogenesis deficits through modulation of TCF4 activity. Previous studies showed that TCF4 dosage affects hippocampus-dependent behaviour [23, 51]. It will be interesting to determine whether deficits in adult neurogenesis contribute to hippocampal dysfunction in *Tcf4* haploinsufficient mice and whether behavioural modifications such as enriched environment can ameliorate *Tcf4* haploinsufficiency associated hippocampus-dependent cognitive deficits in adult mice.

## Conclusion

Our findings suggest that in rodents *Tcf4* haploinsufficiency may have a continuous negative impact on hippocampal function by perturbing the physiological formation of new neurons in the adult dentate gyrus. Moreover, our findings raise the interesting possibility that behavioural interventions may allow to ameliorate a subset of *Tcf4* haploinsufficiency associated neural deficits during adulthood.

## Methods

### Animals and Ethics Statement

All animal experiments were conducted in accordance with the European Communities Council Directive (86/609/EEC) and received ethical approval by the committee for Animal Research of the Bavarian State authorities. The generation of the knockout allele has been described previously [13]. All animals—except for the animals in the enriched environment experiments—were housed in standard cages (size: 37 × 21 × 15 cm) with 3–4 mice per cage under a 12 h light/dark cycle with unlimited access to water and standard rodent food. Mice were housed in the animal facilities of the Helmholtz Center Munich and the Friedrich-Alexander-Universität Erlangen. Animal care was in accordance with institutional guidelines.

Genotyping of the mice was done using PCR and the following primers:

*Tcf4Het*fwd MutTCG TGG TAT CGT TAT GCG CC.

fwd WTCCG ATG ACA GTG ATG ATG GT.

revAAG TTA AGC TGA AGT AAA TAC CCA CA.

lacZ fwdATC ACG ACG CGC TGT ATC.

lacZ revACA TCG GGC AAA TAA TAT CG.

### BrdU injections and Enriched environment

At the age of eight weeks intraperitoneal 5-bromo-2′-deoxyuridine (BrdU) injections were performed twice a day on three consecutive days (0.1 mg BrdU/g body weight, per dose). Animals were sacrificed either three hours (6 control and 6 haploinsufficient mice) after last injection or after additional four weeks (9 control and 5 haploinsufficient mice).

An additional cohort of eight week old *Tcf4* haploinsufficient mice (5 animals) was placed in an enriched environment (EE cage size: 60 × 26 × 33 cm, containing running wheels, toys, tunnels and nest materials). Mice were injected twice intraperitoneal with BrdU on three consecutive days (0.1 mg BrdU/g body weight, per dose). Animals were sacrificed four weeks after the last injection.

### Tissue preparation

Mice were killed using CO_2_ and perfused transcardially with PBS for 5 min (20 ml/min), followed by fixation with 4% paraformaldehyde (PFA) in 0.1 M phosphate buffer (PB), pH 7.4, for 5 min. Brains were postfixed overnight in 4% PFA at 4 °C, followed by dehydration in 30% sucrose in 0.1 M PBS at 4 °C. Brains were cut coronally at 40 µm, using a sliding microtome. Sections were stored at − 20 °C.

### Immunohistochemistry

Free‐floating sections were rinsed six times for 10 min in Tris‐buffered saline (TBS: 1 M Tris–HCL, pH 7.5/0.9% NaCl), and incubated for 72 h at 4 °C with primary antibodies (Table 1) in blocking solution, containing 0.25% Triton X-100 and 3% donkey serum in TBS. Sections were rinsed in TBS six times for 10 min, incubated overnight at 4 °C with fluorochrome‐labelled secondary antibodies (Table 2) diluted in blocking solution, and rinsed in TBS three times for 10 min. Nuclei were counterstained with DAPI (1:10,000 in 1xTBS) for 10 min, followed by three rinses in TBS for 10 min. Sections were mounted on slides and covered with Aqua-Poly/Mount (Polysciences).

**Table 1:** Primary antibodies

Antigen	Host	Manufacturer	Dilution	Catalog number	RRID
BrdU	Rat	Serotec	1:500	OBT0030CX	AB_609566
DCX	Goat	Santa Cruz Biotechnology	1:500	sc-8066	AB_2088494
CALBINDIN	Mouse	Swant	1:300	C9638	AB_2314070
MCM2	Mouse	BD Bioscience	1:500	610,700	AB_2141952
MCM2	Rabbit	Cell Signaling Technology	1:500	4007S	AB_2142134
NESTIN	Mouse	Millipore	1:500	MAB353	AB_94911
PROX1	Rabbit	Chemicon International	1:500	AB5475	AB_177485
TCF4	Rabbit	Abcam	1:500	AB130014	−

**Table 2:** Secondary antibodies

Fluorophore	Epitope	Manufacturer	Dilution	Catalog number	RRID
Alexa488	Anti-Goat	Invitrogen	1:500	A11055	AB_2534102
Alexa488	Anti-Rabbit	Invitrogen	1:500	A21206	AB_2535792
Cy3	Anti-Rat	Jackson	1:500	712–165-153	AB_2340667
Cy3	Anti-Goat	Jackson	1:500	705–165-147	AB_2307351
Cy5	Anti-Mouse	Jackson	1:500	715–175-151	AB_2340820
Cy5	Anti-Rabbit	Jackson	1:500	711–495-152	AB_2315775

For BrdU stainings, slices were first stained for all antigens of interest except for BrdU. Slices were then postfixed in 4% PFA for 10 min at room temperature. Sections were rinsed three times in TBS, incubated in 2 N HCl for 10 min at 37 °C. After two rinses in 0.1 M borate buffer, sections were washed three times with PBS. Detection of BrdU immunoreactivity was conducted as described above.

### Imaging and quantification

For volume, BrdU, and MCM2 quantification, fluorescence signal was detected with an AF6000 Modular Systems Leica fluorescent microscope and documented with a SPOT-CCD camera and the Leica software LAS AF (Version 2.6.0.7266; Leica Microsystems, Wetzlar Germany). For co-localization analyses, fluorescence signal was detected using a Zeiss LSM 780 confocal microscope with four lasers (405, 488, 550, and 633 nm) and × 25 and × 40 objective lens. Images were processed using ImageJ.

For each animal, a series of every 12th section of the dentate gyrus was selected. Volume measurements were performed with ImageJ by tracing the granular zone of dentate gyrus. For BrdU and MCM2 quantification, cells in the granule cell layer and contiguous subgranular zone were counted (52). For co-localization analyses, all BrdU + cells within the granule cell layer and contiguous subgranular zone in at least one section were analysed for expression of DCX or PROX1. A minimum 50 cells per animal were analysed per marker and animal. Statistical analysis was performed with GraphPad Prism (Graphpad Software Inc.), using unpaired two tailed t-test. The data are expressed as mean values ± SEM. Significant differences were assumed at a level of *p* < 0.05.

### Antibodies

See Tables 1 and 2.

## Data Availability

The datasets used and/or analysed during the current study are available from the corresponding author on request.

## Abbreviations

bHLH: Basic Helix-Loop-Helix
BrdU: 5-Bromo-2′-deoxyuridine
EE: Enriched environment
PFA: Paraformaldehyde
PTHS: Pitt-Hopkins-Syndrome
TCF3: Transcription factor 3
TCF4: Transcription factor 4
TCF12: Transcription factor 12
